# Experimental Study of Curing Temperature Effect on Mechanical Performance of Carbon Fiber Composites with Application to Filament Winding Pressure Vessel Design

**DOI:** 10.3390/polym15040982

**Published:** 2023-02-16

**Authors:** Jianguo Liang, Lihua Liu, Zelin Qin, Xiaodong Zhao, Zhi Li, Uwayezu Emmanuel, Jun Feng

**Affiliations:** 1 College of Mechanical and Vehicle Engineering, Taiyuan University of Technology, Taiyuan 030024, China; 2 International Cooperation Division, Taiyuan University of Technology, Taiyuan 030024, China; 3 National Key Laboratory of Transit Physics, Nanjing University of Science and Technology, Nanjing 210094, China

**Keywords:** CFRP, curing temperature, DSC, composite pressure vessel, epoxy resin

## Abstract

During the forming process of carbon fiber composite pressure vessels, the parameters of the curing and forming processes become one of the critical factors affecting the production cost and forming quality. The curing temperature of 4251 A4/B2 epoxy resin is measured in this research, and the effect of curing temperature on the mechanical properties of composite materials for winding is studied, which is finally verified in the test of pressure vessels. First, the actual curing temperature of the epoxy resin is tested and analyzed using differential scanning calorimetry (DSC). Second, under two different curing regimes, the tensile and flexural properties are tested by making pure epoxy resin matrix test pieces, Naval Ordnance Laboratory (NOL) rings, and carbon fiber composite unidirectional plates that affect the overall performance of composite pressure vessels. At the same time, the test results provide reliable process parameters for numerical simulation and manufacturing of pressure vessels. Finally, the filament-wound 35 MPa type III pressure vessel is cured and carried out using a hydraulic burst test. The results show the resin matrix has good fluidity and excellent interface bonding with carbon fiber when the curing temperature is 112 °C. Compared with the results in curing temperature of 100 °C, the tensile strength of the NOL ring reaches 2260.8 MPa, up by 22%. In the 90° direction, the tensile and flexural strengths of the unidirectional plates increase by 68.86% and 37.42%, respectively. In the 0° direction, the tensile and flexural strengths of the unidirectional plates increase by 5.82% and 1.16%, respectively. The pressure vessel bursting form is reasonable and meets the CGH2R standard. The bursting pressure of the vessel is up to 104.4 MPa, which verifies the rationality of the curing regime used in the curing process of the pressure vessel. Based on the results of this paper, the curing temperature affects the fluidity of the epoxy resin, which in turn affects the interfacial bonding properties of the composite, and the forming quality of the wound components and the pressure vessel, ultimately. When using 4251A4/B2 epoxy resin for wet winding pressure vessels, the choice of a 112 °C curing temperature will help improve the vessel’s overall performance. This work could provide reliable experience and insight into the curing process analysis of pressure vessel manufacturing.

## 1. Introduction

Carbon fiber composites have been widely used in aerospace, transportation, military products, and biomedical systems [1,2,3,4,5] because of their light weight, high strength, and high stiffness. In practical engineering applications, it has excellent durability, fatigue resistance, and corrosion resistance compared to traditional steel materials [6,7,8]. Carbon fiber composite pressure vessels, as the main storage method for gas and energy in transportation, highlight the importance of composite material development. However, the performance of carbon fiber composite pressure vessels depends not only on the performance of the component materials and the design of the product winding structure but also on the curing regime of the composite material. With the improvement in carbon fiber properties and the development of various high-performance resin matrices, the study of the factors influencing its curing and forming has become an important direction in the field of carbon fiber composite research based on improving production efficiency and overall performance.

As a hot spot in the research field of carbon fiber composites, many scholars mainly consider the influence of winding process parameters in their design and manufacture. Many studies have focused on winding trajectory, winding sequence, winding angle, liner optimization, nondestructive testing [9,10,11], and the evaluation of winding layer forming quality, fatigue life, and burst pressure [12,13]. However, the curing and molding process parameters are lacking in-depth studies, and the degree of cure (DOC) is often improved by changing the curing and forming method, which improves the composite material performance [14,15,16].

The DOC depends on the physical properties of the resin matrix (viscosity, specific heat, melting point, and thermal conductivity) [17,18,19], which ultimately determine the mechanical properties of the composite. Curing kinetics can reflect the relationship of the curing rate to the DOC, curing temperature, and curing time. Therefore, when studying issues of pressure vessel curing and forming, the curing kinetics of the specific epoxy resin matrix need to be determined first.

The curing kinetics of epoxy resin systems have been widely investigated, with studies focusing on the kinetics of epoxy resins with different curing agents [20,21,22], the properties of modified versus unmodified epoxy resins [23,24,25], and the effects of fillers, curing temperature and curing pressure on the kinetics of different epoxy resin-reinforced composites [26,27,28,29,30,31,32]. There are various methods to study curing kinetics, among which DSC is the most commonly used method [33,34]. The relationship of the curing rate to DOC, curing temperature, and curing time can be obtained using DSC analysis of the epoxy resin system by isothermal or non-isothermal methods. The experimental results are analyzed and fitted with several classical kinetic models to obtain the kinetic parameters [35,36,37] and thus accurately predict the kinetic behavior of the reaction system.

Curing temperature has a significant effect on the curing process, and a large number of researchers have investigated the effect of curing temperature on the properties of composite materials. Ma et al. [38] investigated the effect of the no-pressure curing temperature on the flexural properties of 2D-T700/E44 composites, which were prepared at six different no-pressure curing temperatures. The results showed that either too high or too low unpressurized curing temperatures were not favorable for the preparation of the composites. Li et al. [39] investigated the interfacial shear strength and fracture toughness of the carbon fiber–TiO${}_{2}$ system at different curing temperatures, and the results showed that the interfacial shear strength and fracture toughness of the carbon fiber–TiO${}_{2}$ system was significantly improved after high-temperature curing, which could reach 8.34 MPa and 1.68 J/M${}^{2}$. Man et al. [40] investigated the effect of three groups of curing temperatures, “without post-curing,” “post-cured at 80 °C for 6 h,” and “post-cured at 120 °C for 3 h,” on the performance of carbon fiber reinforced polymers (CFRP) and glass fiber reinforced polymer (GFRP). The results showed that the hardness and flexural strength of both CFRP and GFRP were improved, but the mechanical strength of CFRP was not significantly improved, and that of GFRP was slightly improved. Ismail et al. [41] investigated the effect of curing temperature on the mechanical properties of bio-phenolic/epoxy polymer blends. The results showed that the tensile strength of the blends was proportional to the curing temperature, and the tensile modulus was inversely proportional to the curing temperature. Koushyar et al. [42,43] investigated the effects of autoclave temperature and pressure on the porosity, compressive strength, and short beam shear strength (SBS strength) of carbon fiber/epoxy composites. The results showed that the curing degree and glass transition temperature were closely related to the hot press tank temperature. The SBS strength of the composites was inversely proportional to the autoclave temperature, and their SBS strength was about 10% lower for specimens cured at 149 °C than for those cured at 160 °C to 182 °C.

These works mainly focus on composite laminates reinforced by different resin systems; however, there are few reports on the curing process parameters of pressure vessels. The purpose of this study is to investigate the effect of the curing temperature of 4251A4/B2 epoxy resin on the mechanical properties of composites for winding and to select a suitable curing regime for pressure vessel curing and molding. The curing temperature of the epoxy resin (112 °C) is solved using DSC, and the curing kinetic equations of the epoxy resin are derived. Based on this, the tensile, flexural, and shear properties of the pure epoxy resin matrix, NOL rings and composite unidirectional plates are compared for two different curing regimes. The accurate derivation of the curing kinetic parameters provides a theoretical basis for modeling pressure vessel curing and molding, and the test results provide process parameters for numerical simulation and actual winding of pressure vessels. Finally, a hydraulic burst test is conducted on the wound 35 MPa pressure vessel to verify the rationality of the curing regime. The evaluation of the overall performance of the pressure vessel and the microstructure state after rupture demonstrate the applicability of the curing regime.

## 2. Experimental Program

### 2.1. Material

The 4251A4/B2 epoxy resin is provided by Guangdong Bohui New Material Technology Co., Ltd., Zhaoqing, Guangdong, China and the T700SC-12K carbon fiber is provided by GuangWei Composite Co., Ltd., Weihai, Shandong, China. The material’s main parameters are shown in Table 1 and Table 2.

### 2.2. Pure Epoxy Resin Matrix Preparation

**Figure 1:**
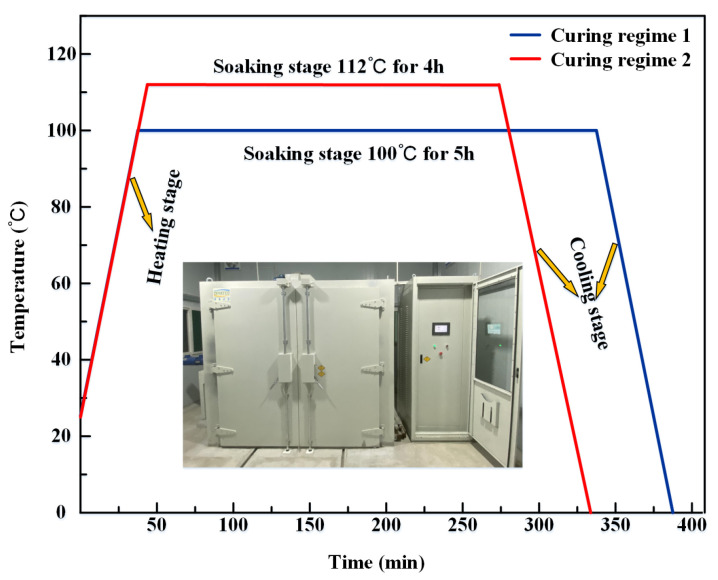
Curing regimes.

First, the 4251A4/B2 epoxy resin was weighed a certain amount of epoxy resin A4 and curing agent B2, respectively, and material A4 and B2 were mixed according to the mass ratio of 100:90, stirred for 10 min at the same time, and left to stand for 20 min to eliminate the air bubbles after mixing well. Second, the mixed epoxy resin was cast in the polytetrafluoroethylene (PTFE) mold. The casting was put into the vacuum drying oven to finish curing according to two different curing regimes, as shown in Figure 1.

### 2.3. Preparation of Composite NOL Rings and Unidirectional Plates

**Figure 2:**
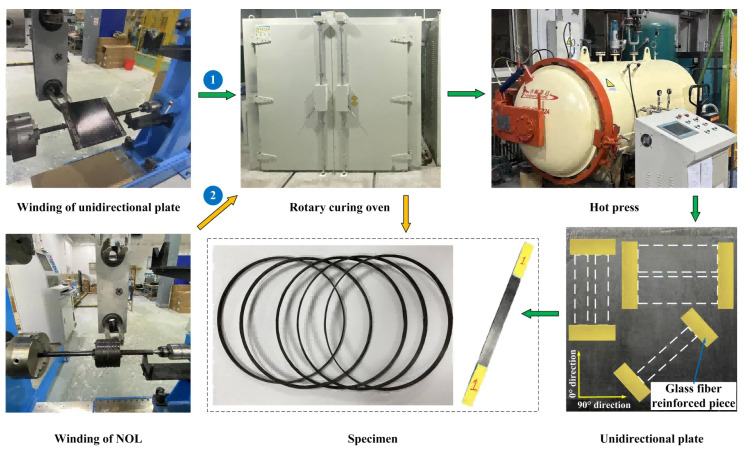
NOL ring and unidirectional plate preparation process.

The wet winding method is adopted. First, the mixed epoxy resin was left to defoam and poured into the dipping tank of the winding machine. Second, the same winding process parameters (such as winding tension, winding rate, and resin temperature) were set so as to ensure the same fiber volume fraction of the wound parts. The NOL ring and unidirectional plate preparation process are shown in Figure 2.

To ensure the reliability of the experimental test data results, the same batches of carbon fiber and epoxy resin were used for the test materials. The winding pressure vessel was designed to verify the reasonableness and excellent characteristics of the curing temperature and curing regime. With reference to the standard CGH2R [44], the main design indexes of the pressure vessel are as follows: working pressure of 35 MPa, minimum design burst pressure of 82.25 (2.35 times the working pressure), and the number of fatigue life ≥ 11,000. The pressure vessel was wound by spiral and circumferential winding, and the design of the winding angle and thickness refers to the grid theory [45]. The lay-up sequence was selected as [$90_{2}^{\circ }/\pm 11_{4}^{\circ }/90_{4}^{\circ }/\pm 11_{2}^{\circ }/90_{4}^{\circ }/\pm 11_{2}^{\circ }/90_{4}^{\circ }/\pm 15_{2}^{\circ }/90_{4}^{\circ }/\pm 15_{2}^{\circ }$], and the pressure vessel aluminum lining was wound according to the winding sequence. To ensure the validity and comparability of the experimental results, the winding tension of the NOL ring, unidirectional plate, and composite pressure vessel was designed to be 21 N, the winding rate was 10–20 rad/min, and the temperature of the dipping tank was 35–40 °C.

### 2.4. Thermal Analysis

A DSC provided by Beijing Hengjiu Experimental Equipment Co., Ltd., Beijing, China was used for the dynamic heating test, which was performed on the epoxy resin in a flowing argon gas (flow rate of 50 mL/min) environment. An amount of 10 mg of well-mixed epoxy resin was weighed and put into an aluminum crucible with a sealed lid. The heating range was from room temperature to 350 °C, and the heating rates were 5 °C/min, 10 °C/min, 15 °C/min, and 20 °C/min.

### 2.5. Mechanical Performance Test

According to Figure 2, the NOL ring and the unidirectional plate were produced. The test standard of pure epoxy resin matrix was GB/T 2567-2021 [46]. The composite NOL ring needed to be polished to a flat surface before testing, and its test is fixed in a unique NOL ring stretching fixture and refers to standard GB/T 1458-2008 [47]. The standard sample of composite unidirectional plate test is cut at different angles regarding 0° fiber direction by waterjet cutting equipment to ensure the high-quality shape and size of the test specimens. Referring to the test standard ASTM D3039 [48], ASTM D3518 [49], glass fiber reinforcement sheets were pasted on both ends of the tensile and shear specimen, and the size of the tensile specimen was 250 mm × 15 mm × 2 mm, and the size of the shear specimen was 250 mm × 25 mm × 2 mm. The flexural specimen needs to ensure that the span was 32 times the thickness, the test standard was ASTM D790 [50], and the specimen size was 80 mm × 12.5 mm × 2 mm. The above standard specimens were tested using an electronic universal material testing machine (INSTRON5969, ITW Group Instron, Shanghai, China).

### 2.6. Material Scanning Electron Microscopy (SEM) Testing

In this paper, ZEISS Gemini SEM 300 was used to observe the specimens of hydrogen storage vessels after hydraulic burst tests, specifically the composite layers of the dome section, transition section, and cylinder section. The test started by cutting the composite layers from the selected parts to a size close to 40 mm × 10 mm × 30 mm and then cleaning the fracture of the composite layer damage using anhydrous ethanol without damaging the observed end surfaces. Test observation specimens need to be spray-gold treated for 60 s, and multiples of the dome section, transition section, and cylinder section taken 300 times, 1000 times, and 500 times in turn.

## 3. Results and Discussion

### 3.1. Curing Kinetics Analysis

#### 3.1.1. Differential Scanning Calorimetry Curve Analysis

The curing kinetics of epoxy resin 4251A4/B2 were investigated using the dynamic heating method using DSC instrumental tests. The onset temperature ($T_{i}$), peak temperature ($T_{p}$), and termination temperature ($T_{f}$) of the curing exothermic peak of the epoxy resin at different heating rates were obtained based on DSC diagrams.

**Figure 3:**
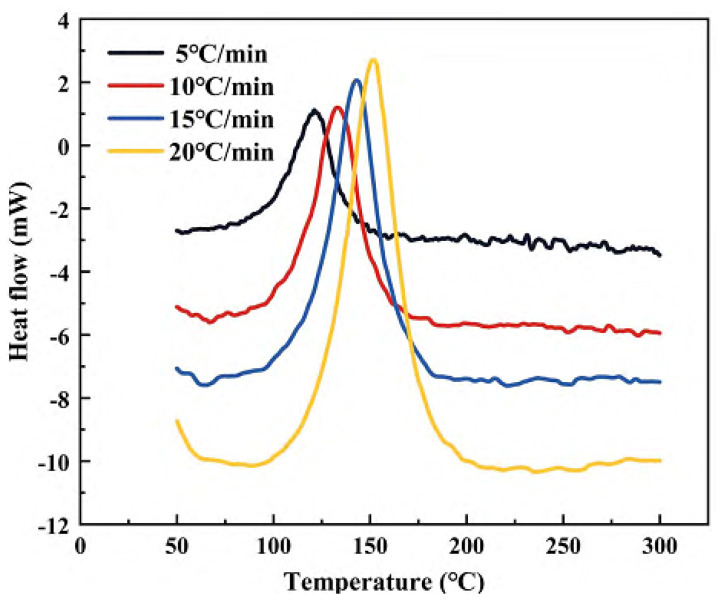
Dynamic DSC thermal analysis curves of epoxy resin at different temperature rise rates.

As seen in Figure 3, the following conclusions can be drawn from the DSC diagrams of the epoxy resin systems.

(1) The onset temperature, peak temperature, and termination temperature of the resin system increase as the heating rate rises;
(2) The appearance of the exothermic peak of the resin system increases with the rate of temperature rise and moves to the right, that is, in the direction of high temperature;
(3) With the increase in the heating rate, the curing temperature rises, the curing rate increases, and the curing time is shortened.

**Figure 4:**
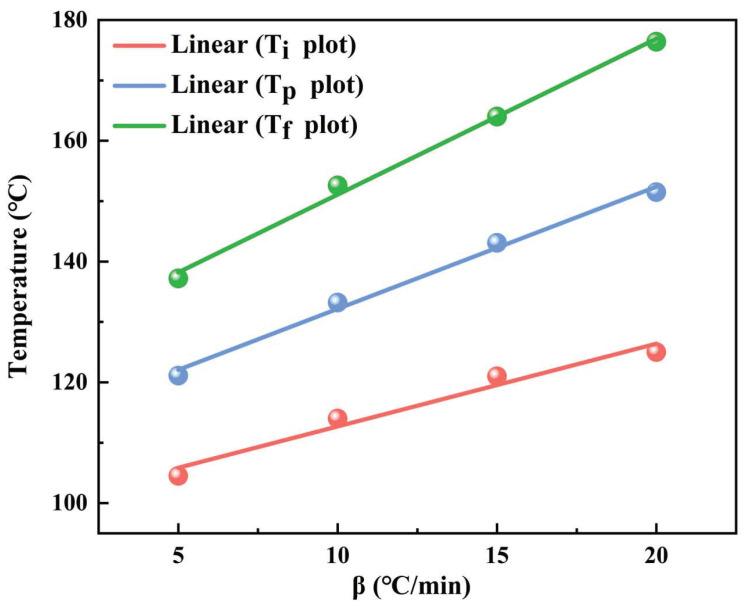
Temperature extrapolation curves at different heating rates.

As seen from Figure 4, according to the least square method, three temperature values ($T_{i}$,$T_{p}$,$T_{f}$) can be obtained when the heating rate is zero. The extrapolated temperatures are the theoretical start, peak, and end temperatures of the epoxy resin system, which will be used as a reference for resetting the temperature values of the curing conditions. Table 3 shows the specific values of $T_{i}$, $T_{p}$, and $T_{f}$ at different heating rates and their extrapolation results.

#### 3.1.2. Curing Kinetics Parameters Analysis

The curing kinetic model mainly includes the phenomenological model [51] and the mechanism model [52]. The mechanism model is more accurate than the phenomenological model, but it is difficult to model because it considers the kinetic mechanism in the whole reaction process. Therefore, the phenomenological model is widely used in the curing process of composite materials.

The phenomenological model expression is

(1) dα/dt=K(T)f(α)

where dα/dt is the curing reaction rate, *T* is the absolute temperature, f(α) is the curing mechanism function, and K(T) is the reaction rate constant, following the Arrhenius equation, as shown in Equation (2).

(2) $$K\left(T\right)=Aexp\left(-E_{a}/RT\right)$$

where *A* is the pre-exponential factor, $E_{a}$ is the activation energy, and *R* is the universal gas constant.

There are two kinds of curing mechanism functions: the n-order reaction model and the autocatalytic reaction model. The n-order reaction model is the simplest model to describe the curing reaction.

(3) $$f\left(\alpha \right)={\left(1-\alpha \right)}^{n}$$

(4) $$f\left(\alpha \right)=\alpha^{m}{\left(1-\alpha \right)}^{n}$$

where *n* and *m* are the reaction orders, the epoxy resin model in this paper can be described by Equation (5).

(5) $$d\alpha /dt=Aexp\left(-E_{a}/RT\right)f\left(\alpha \right)$$

The kinetic reaction parameters (activation energy $E_{a}$, pre-exponential factor *A*, and reaction model f(α)) are determined using a model-free estimation method designed by Kissinger [53] and Ozawa [54,55] based on the following equations.

Kissinger equation:

(6) $$\frac{d\ln(\beta /T_{p}^{2})}{dT_{p}^{-1}}=-\frac{E_{a}}{R}$$

Transforming the two sides of the above equation to Equation (7).

(7) $$\ln\left(\frac{\beta }{T_{p}^{2}}\right)=\ln\left(\frac{AR}{E_{a}}\right)-\frac{E_{a}}{RT_{p}}$$

Ozawa equation:

(8) $$\frac{d\ln\beta }{d1/T_{p}}=-1.052\frac{E_{a}}{R}$$

Transforming the two sides of the above equation to Equation (9).

(9) $$\frac{\ln\beta }{1/T_{p}}=-\frac{1.052}{R}E_{a}$$

where β is the heating rate, $E_{a}$ is the activation energy obtained with the Kissinger and Ozawa equations, and $T_{p}$ is the peak temperature.

**Figure 5:**
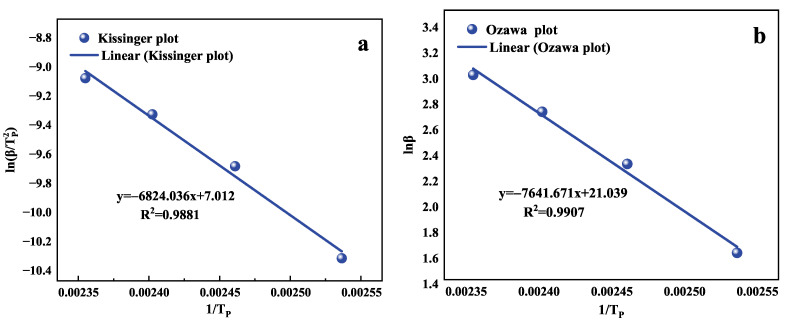
(**a**) The curve of activation energy of the curing reaction was determined by the Kissinger method; (**b**) The curve of activation energy of the curing reaction was determined by the Ozawa method.

The apparent activation energy can be obtained by fitting the linear relationship between Kissinger’s $\ln(\beta /T_{p}^{2})$ versus $1/T_{p}$ and Ozawa’s lnβ versus $1/T_{p}$ according to the transformed Kissinger and Ozawa equations, as shown in Figure 5. The apparent activation energy obtained using Kissinger’s method and Ozawa’s method is higher than that of the epoxy resin 4251A4/B2, but the difference between the two is small, only 3.657 kg/mol. The average value of 58.564 kg/mol is obtained as the apparent activation energy of the epoxy resin 4251A4/B2.

Crane [56] further derives the model proposed by Kissinger to obtain the equation.

(10) $$\frac{d\ln\beta }{d1/T_{p}}=-\left(\frac{E_{a}}{nR}+2T_{p}\right)$$

when $\frac{E_{a}}{nR}$ is much larger than $2T_{p}$, $2T_{p}$ can be neglected.

According to the literature, a linear fit to $\ln(\beta /T_{p}^{2})$ versus $1/T_{p}$ is performed to obtain the intercept, which is substituted into $E_{a1}$ to obtain *A* as 7.57 * 10${}^{6}$ min${}^{-1}$. The slope is obtained by linearly fitting lnβ versus $1/T_{p}$ using the Crane equation and substituting $E_{a1}$ so as to get *n* as 0.89. The specific parameters are shown in Table 4.

Thus, the n-order curing kinetic model equation of 4251A4/B2 is established.

(11) $$\frac{d\alpha }{dt}=7.57*10^{6}exp\left(-6824.04/T\right){\left(1-\alpha \right)}^{0.89}$$

#### 3.1.3. Determination of Curing Temperature

The curing regime is generally divided into two-step and three-step temperature gradients. The curing step temperature is divided into pre-curing temperature, curing temperature, and post-curing temperature. According to the $T_{i}$, $T_{p}$, and $T_{f}$ measured by dynamic heating experiments, the pre-curing temperature is set as the starting temperature, the theoretical curing temperature is set as the peak temperature, and the post-curing temperature is set as the termination temperature. However, due to the small differences among the three values, the curing temperature setting process chooses $T_{p}$ as the actual curing temperature, that is, 112 °C as the actual curing temperature. It is recommended that the 4251A4/B2 epoxy resin curing temperature is 100 °C × 5 h. The two curing systems selected for the epoxy resin are shown in Figure 1.

### 3.2. Effect of Curing Temperature on the Mechanical Properties of Pure Epoxy Resin Matrix

**Figure 6:**
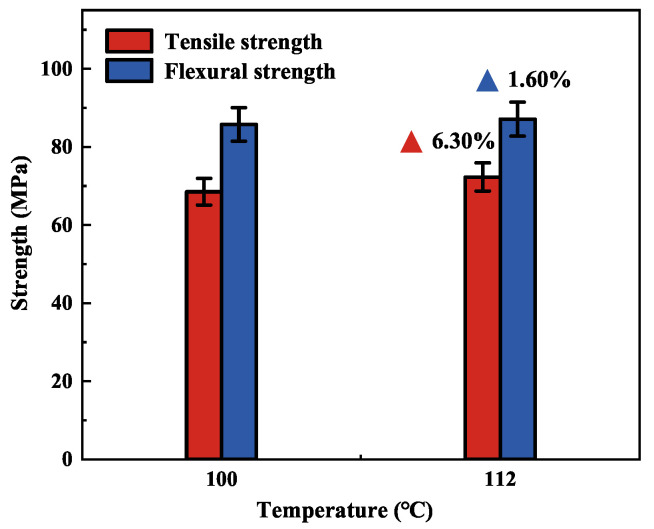
Temperature extrapolation curve at different heating rates.

The pure epoxy resin matrix is cured at 100 °C for 5 h and 112 °C for 4 h. As seen in Figure 6, the tensile strength of the pure epoxy resin matrix is improved by 6.30%, and the flexural performance is improved by 1.60% under two different curing temperatures. The results show that the change in curing temperature from 100 °C to 112 °C resulted in a slight increase in the fracture resistance of the pure epoxy resin matrix, and the flexural resistance remained the same. At the theoretical curing temperature, although the mechanical properties of the pure epoxy resin matrix are lower, this has a positive effect on the overall performance of the composite material when combined with fibers.

### 3.3. Effect of Curing Temperature on Mechanical Properties of Composites

For the structural design and fabrication of pressure vessels using laminate theory, it is necessary to obtain the tensile strength and modulus of carbon fiber composites in the 0°, 90°, and shear directions of the material. However, NOL rings and unidirectional plates are two types of unidirectional fiber winding specimens commonly used to test the strength and modulus of carbon fiber composites, and the strength and modulus of NOL rings in the 0° direction can provide more realistic process strength data for pressure vessels because of their similar winding and load-bearing conditions. According to the test results of standard specimens of NOL ring and unidirectional plate in each direction, it can reflect the interfacial wettability between fiber and resin matrix, bond strength, and its ability to transfer stress under stress conditions to a certain extent.

In the process of combining epoxy resin with carbon fiber, the epoxy resin itself is mixed with the curing agent and reaches a uniformly distributed state through a mutual contact and winding of molecules. The epoxy group with the active hydrogen in the amino group of the curing agent undergoes a condensation polymerization reaction to form a high molecular weight epoxy compound. The change of epoxy resin from a liquid to a solid state under the influence of temperature is caused by chemical changes between molecules, and the degree of reaction of gradual polymerization directly affects the end-use properties of the cured product. The crosslinking density is an indicator of the degree of the polymerization reaction, which is crucial to the final performance of the epoxy resin.

**Figure 7:**
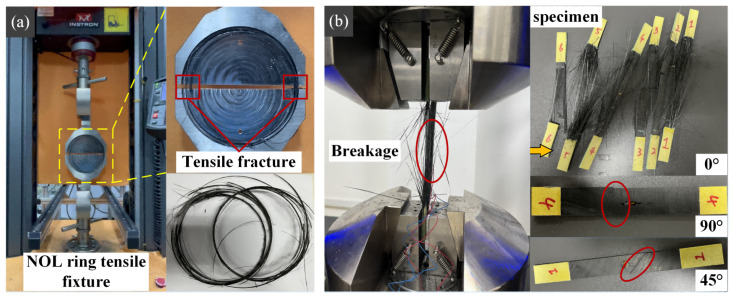
(**a**) NOL ring tensile test; (**b**) Unidirectional plate tensile test.

The mechanical properties of the composite NOL rings are listed in Table 5, where “4251-100” indicates that the curing temperature of the epoxy resin in the carbon fiber composite is 100 °C. The tensile strength of the composite NOL rings increased by 22% when the curing temperature was changed from 100 °C to 112 °C. Figure 7 shows the form of tensile fracture of the unidirectional plate. According to Figure 7, the failure form of NOL shows the fracture form is reasonable. The gradual delamination of the NOL rings occurred with an increasing tensile load. Compared with the specimen when the curing temperature was 100 °C, the delamination of NOL occurred later at 112 °C. Therefore, it can be concluded to a certain extent that the interfacial bonding ability of epoxy resin and fiber in the composites at 112 °C is better enhanced, thus improving the overall load-carrying capacity. After delamination, under the maximum tensile load, the fibers at both ends of the NOL exerted ultimate strength while producing fracture.

**Figure 8:**
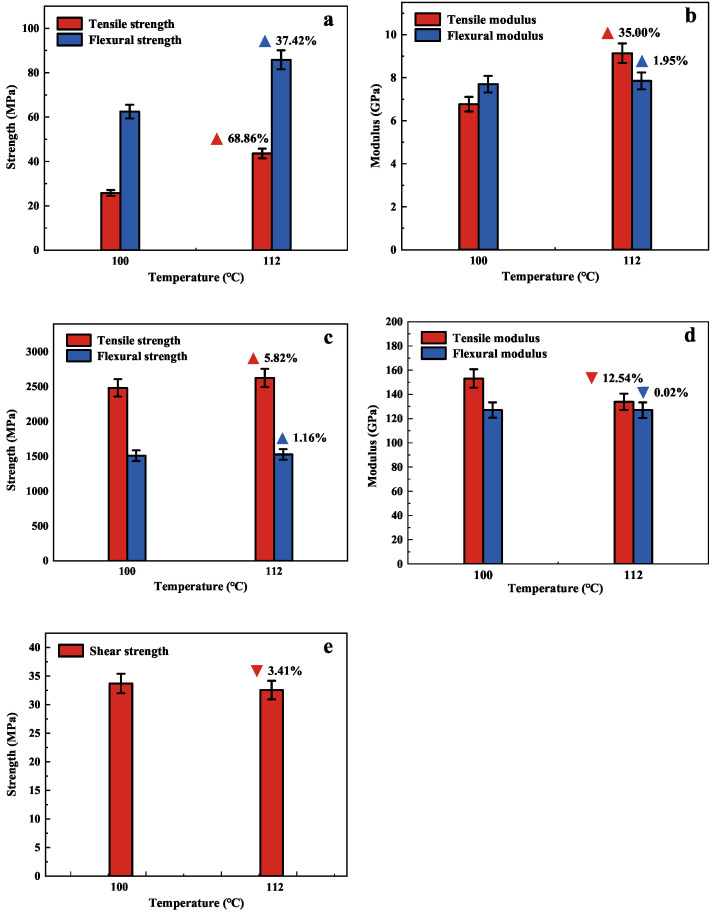
(**a**) Tensile and Flexural strength in 90° direction; (**b**) Tensile and Flexural modulus in 90° direction; (**c**) Tensile and Flexural strength in 0° direction; (**d**) Tensile and Flexural modulus in 0° direction; (**e**) Shear strength in 45° direction.

**Figure 9:**
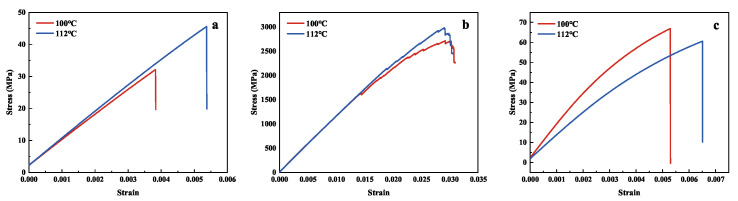
(**a**) Tensile stress-strain in 90° direction; (**b**) Tensile stress-strain in 0° direction; (**c**) Tensile stress-strain in 45° direction.

Figure 8 shows the mechanical properties of the composite unidirectional panels at different curing temperatures. Figure 9 shows the tensile stress-strain curves of the composite unidirectional panels in different directions. The tensile strength, flexural strength, and tensile modulus of the composite unidirectional plate are significantly improved in the 90° direction, and the tensile strength increased by 68.86% and flexural strength increased by 37.42%, but the flexural modulus changed less. In the 0° direction, the tensile strength increased by 5.82%, but the tensile modulus decreased by 12.54%. However, the flexural strength, flexural modulus, and shear strength in the 45° direction showed little change, and the test results are in good agreement with Ismail’s [41] results.

The improvement of mechanical properties of NOL rings and composite unidirectional plates is due to the fact that the epoxy resin is the first to reach the exothermic state at 112 °C, and the molecules reach the temperature at which crosslinking reaction occurs. The accelerated movement between molecules and the intensification of the crosslinking reaction increases the crosslinking bonds, resulting in a higher crosslinking density and more molecular chains, which eventually lead to the improvement of tensile strength. In the 0° direction, the polymer is formed during the movement of epoxy resin molecules. Due to the large size of the polymer, the molecular movement space is blocked, and the free volume fraction within the epoxy resin system decreases, which eventually leads to a decreasing trend of the elastic modulus. The results show that the mechanical properties of the carbon fiber composites are substantially improved, so it is proved that the curing temperature derived from the extrapolation method is reasonable in this paper. In addition, the obtained curing regime is also more reasonable to be applied to manufacturing pressure vessels.

### 3.4. Burst Pressure Results of Composite Pressure Vessel

Based on the fixed epoxy resin model, the new curing temperature (112 °C) is obtained using the extrapolation method in the curing kinetics analysis section of this paper. Compared with the curing temperature of 100 °C, the mechanical properties of the NOL ring and unidirectional plate in all directions are better developed at 112 °C. Therefore, it is applied to the pressure vessel curing process to verify the rationality and advantages of the curing regime derived with extrapolation.

**Figure 10:**
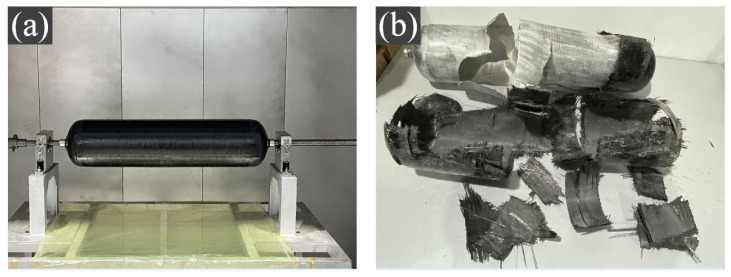
(**a**) Pressure vessel curing; (**b**) Hydraulic burst test.

The pressure vessel is designed according to the 35 Mpa standard CGH2R and alternatively wound in spiral and circumferential winding methods. A total of 14 layers of spiral and circumferential winding (28 layers for a single layer) are cured using a 112 °C × 4 h rotational curing regime, as shown in Figure 10a. The cured pressure vessel is put under a hydraulic burst test according to standard ISO19881 [57], with a pressure loading rate of 0.35 MPa/s. The experimental process fully complied with the standard.

The pressure vessel bursting results are shown in Figure 10b. The burst pressure reaches 104.4 MPa, 26.93% higher than the minimum design burst pressure. From the burst test results, it can be shown that the burst location is located in the cylinder part and meets the standard design. The pressure vessel liner is deformed and bulged on the side near the transition section, and the fiber layer is fractured from the main load-bearing circumferential fiber layer. At the place of the liner rupture in the middle of the cylinder part, the circumferential (90°) fibers first reach the maximum tensile strength in the fiber direction, so a large area of fiber fracture occurs. As the load gradually increases, the first failure before the fiber-bearing fracture is the fracture of the resin matrix and the fiber delamination phenomenon. Due to the high bursting pressure, the first fiber winding layer, which is the first to start delamination, may be completely separated from the lining surface as the fibers fracture.

**Figure 11:**
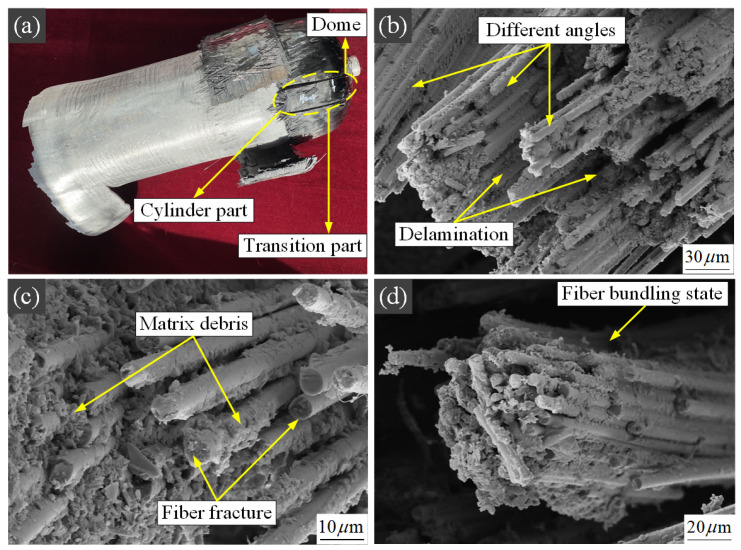
(**a**) SEM observation position after burst test; (**b**) SEM observation of dome part; (**c**) SEM observation of transition part; (**d**) SEM observation of cylinder part.

To investigate the overall properties and failure state of the epoxy resin matrix in the curing regime derived from the extrapolation method, this paper uses the SEM observation and analysis of a section of composite layer after the hydraulic burst test performed using the ZEISS Gemini SEM 300. Considering the integrity of the test, three fiber layers of the dome part, transition part, and cylinder part are observed, respectively, and the observation results are shown in Figure 11a below.

The pressure vessel dome part generally consists of spiral wound fiber layers at different angles to bear the internal pressure load, as shown in Figure 11b. With the increase of load up to the bursting pressure, delamination, and fiber fracture occurs in the fiber layers at different angles. From the figure, it can be seen that although there are many delaminations, there is no significant debonding of carbon fibers from the resin matrix. After delamination, the fibers still appear to be bundled under the action of the resin matrix. Therefore, the fibers and the resin matrix can play a more powerful connecting role with excellent interface bonding performance under the 112 °C curing regime. From the micro perspective, it is verified that good bonding performance is essential to the overall performance of the dome part of the pressure vessel.

As a complex part of the pressure vessel geometry, the structural state of the transition part is crucial in the bearing process. Excessive matrix fracture and delamination can lead to a sudden drop in partial load-bearing performance, which may result in early rupture failure of the pressure vessel at the transition part; a similar analysis and discussion were presented in the research work of Nebes [9]. As shown in Figure 11c, a small amount of fracture of the fiber bundle occurs, and there are residual bonded resin matrices on the fractured fibers, while most of the fibers remain connected to the resin matrix and continue to bear the load. Such a damage mechanism in the carbon fiber composite layers of the transition part is more favorable to the load-bearing of the vessel. Therefore, this is an indirect indication of the excellent curing regime, which maximizes the load-bearing performance of the fiber composite and avoids early failure of the pressure vessel in the transition part.

The cylinder part of pressure vessels has both circumferential and spiral fiber winding layer bearings, but the circumferential fiber winding layer bears more load. As shown in Figure 11d, this paper focuses on observing the circumferential fibers of the composite material in the cylinder part. As the most critical load-bearing part of the pressure vessel, the fractured circumferential fiber bundles still maintain good bonding, while the resin attached to the fiber surface is uniform and dense, which has a positive effect on the quality state of the fiber composite layer with low porosity and few defects in the cylinder section. Thus, this fully reflects the excellent fluidity of the resin at 112 °C curing temperature, which results in better performance of the bonding interface between the fibers and the resin matrix. The results of the burst pressure of the pressure vessel show the importance of the influence of curing temperature on the mechanical properties of carbon fiber composites. In addition, the microstructure states of different parts after the burst can show that the curing regime derived in this paper is reasonable and can improve the overall performance of the pressure vessel.

## 4. Conclusions

In this study, the curing temperature of 4251A4/B2 epoxy resin and its kinetic parameters are derived based on DSC experiments. By comparing the mechanical properties of the pure epoxy resin matrix, NOL ring, and composite unidirectional plate in two curing regimes, the curing temperature obtained based on the extrapolation method is proved to be reasonable, and this corresponding curing regime is applied to a 35 MPa pressure vessel. The following conclusions are drawn:

(1) Compared to 100 °C, the tensile strength of 4251A4/B2 pure epoxy resin matrix at a 112 °C curing temperature is improved by 6.30%, and the flexural strength can still maintain the original level. It shows that the curing temperature of 112 °C positively affects the improvement of the overall properties of epoxy resin used in composites.
(2) The tensile and flexural strength of the composites for winding were improved using a curing regime of 112 °C. The tensile strength of NOL rings was enhanced by 22%. The tensile strength and flexural strength of the unidirectional plates increased by 68.86% and 37.42%, respectively, in the 90° direction, and the tensile strength in the 0° direction increased by 5.82%. The application of this curing regime provides a strong guarantee for the improvement of composite materials and overall performance in hydrogen storage vessels.
(3) The 35 MPa pressure vessel is cured and molded at 112 °C, and its actual burst pressure reaches 104.4 MPa, 26.93% higher than the minimum design burst pressure. At 112 °C, the resin fluidity in the pressure vessel is improved, and the interfacial bonding between the resin matrix and the fibers is enhanced, which ultimately leads to the good overall performance of the pressure vessel. It shows that the selection of the curing regime in the molding process of the pressure vessel is an essential factor in the overall mechanical properties.
(4) The non-isothermal kinetic method is used to analyze the curing kinetics of 4251A4/B2 epoxy resin, and the activation energy values calculated using the Kissinger method and Ozawa method are 56.735 kJ/mol and 60.392 kJ/mol, respectively. Its n-order reaction model will be used in pressure vessel curing molding simulation.
(5) The curing temperature is a crucial factor affecting the curing process. In applying 4251A4/B2 epoxy resin wet-forming composite materials and pressure vessels, the curing temperature of 112 °C is a curing parameter that cannot be ignored to improve the overall performance of the structure.

In the future, the influence of heating and cooling rates and rotation rates on the overall performance of pressure vessels will be further studied on the basis of this paper. The effect of curing temperature will be considered in the pressure vessel curing simulation to obtain more accurate prediction results.

## Figures and Tables

**Table 1:** Physicochemical properties of epoxy resin.

Density (g/mL)	Viscosity (mPa·s)	Gel Time (h)	Gel Time (s)
1.47	1400–1500	>12	80–120

**Table 2:** The main parameters of carbon fiber.

Tensile Strength (MPa)	Linear Density (g/km)	Volume Density (g/cm${}^{3}$)	Elongation (%)	Diameter (μm)
4900	800	1.8	2.1	7

**Table 3:** Specific values and extrapolation results of $T_{i}$, $T_{p}$, and $T_{f}$ at different heating rates.

Heating Rate (°C/min)	$T_{i}$ (°C)	$T_{p}$ (°C)	$T_{f}$ (°C)
5	104.5	121.1	137.2
10	114.0	133.2	152.3
15	121.0	143.1	164.0
20	125.0	151.5	176.4
0	99.0	112.0	125.0

**Table 4:** Kinetic parameters of the 4251A4/B2 epoxy resin system.

Model	$E_{a1}$ (kg/mol)	$E_{a2}$ (kg/mol)	$E_{a}$ (kg/mol)	A (min${}^{-1}$)	*n*
4251A4/B2	56.735	60.392	58.564	7.57 × 10${}^{6}$	0.89

**Table 5:** Tensile properties of NOL rings at different curing temperatures.

Curing Temperature	4251-100	4251-112
Tensile strength (MPa)	1852.37	2260.80

## Data Availability

Not applicable.
